# Genetic hearing loss: the journey of discovery to destination – how close are we to therapy?

**DOI:** 10.1002/mgg3.260

**Published:** 2016-11-21

**Authors:** Arti Pandya

**Affiliations:** ^1^ Division of Genetics and MetabolismDepartment of PediatricsUniversity of North CarolinaChapel HillNorth Carolina

## Introduction

Hearing loss (HL) is an extremely common neurosensory deficit with a heterogeneous etiology including environmental and genetic causes. The incidence of profound sensorineural HL in the United States is 186 per 100,000 births (Morton and Nance 2006). In developed nations, more than 60% of individuals affected with HL have a genetic etiology that can be classified by the mode of inheritance and the presence or absence of other clinical characteristics that permits the diagnosis of specific syndromes associated with HL (Willems et al. 2000, Tekin et al. 2001; Nance 2003). The vast majority of individuals (70%) have nonsyndromic hearing loss (NSHL) which can be inherited as a recessive (80%), dominant (15%), X‐linked or mitochondrial trait (5%). In 30 % of individuals with HL, one may identify a recognizable pattern or syndrome of which nearly 400 forms have been described (Toriello and Smith 2013, Oxford Univ. Press). Among syndromic forms, a few such as Pendred syndrome (Reardon et al. 1997), Usher Syndrome (Petit 2001), or Waardenburg syndrome (Nance 2003; Schultz 2006) occur with a frequency of 1–4% among the deaf.

As suggested by analysis of large collections of family data, genetic heterogeneity was expected with HL (Nance and Pandya 2002). As the loci were identified, they were numbered chronologically, and cataloged where DFNB represents recessive inheritance, DFNA represents postlingual dominant forms, and DFN represents X‐linked loci. An updated list is maintained at the Hereditary Hearing Loss homepage with loci mapped, genes identified at the locus and references (Hereditary Hearing Loss home page: http://hereditaryhearingloss.org/). In view of our knowledge that a large number of genes are involved with HL, and that mutations in each gene would account for a small proportion of all HL, the discovery that mutations in a single gene *GJB2* (DFNB1 locus), encoding connexin 26 (Cx26), accounted for a large proportion of recessive NSHL, came as an unexpected surprise (Kelsell et al. 1997; Pandya et al. 2003). Mutations in Cx26 are the most frequent form of nonsyndromic deafness and its contribution to deafness exceeds 60% in some Western populations (Pandya et al. 2003; Azaiez et al. 2006). Docking of hexameric hemiconnexins on the adjacent surface of two cells forms a complete gap junction which is critical to the flow of ions, especially potassium ions, in the inner ear (Steel and Bussoli 1999). More than 100 mutations of the connexin 26 gene have been reported with some common ethnic specific variants including the 35delG mutation in Caucasians, the 167delT allele in Ashkenazi Jews, and the 235delC allele in Asians (Connexin – Deafness Homepage: http://davinci.crg.es/deafness/). Interestingly, del Castillo et al. (2002) reported a 342 kb deletion spanning the connexin 30 (Cx30) locus, in individuals heterozygous for Cx26, as the cause of their HL. The Cx26 and Cx30 loci are located on 13q11 region within 40 kb of each other; however, the Cx26 locus is not involved in the deletion. Thus, it is unclear if the hearing loss in individuals carrying a mutation in Cx26 and a deletion of Cx30 in trans occurs due to haploinsufficeincy of Cx30 or whether the deletion of Cx30 perturbs the expression of the adjacent normal Cx26 (Rodriguez‐Paris and Schrijver 2009).

Since the identification of GJB2 as a major cause for nonsyndromic autosomal recessive HL, there has been tremendous progress in elucidating the genetic etiology for both nonsyndromic and syndromic forms of HL, with nearly 1% of the genome coding for transcripts determined to be important in the development and functioning of the hearing apparatus.

## Molecular Advances in Understanding Genetic Etiology of Hearing Loss

Despite the remarkable genetic heterogeneity known with hearing loss, tremendous success in mapping and identifying the several hundred loci for HL can be attributed to several factors and advances occurring in parallel. First, innovative approaches and methods to map genetic loci in consanguineous families or population isolates was instrumental in mapping genes for various nonsyndromic AR forms of HL such as the DFNB3 (Guilford et al. 1994; Friedman et al. 1995). The initiation of large‐scale mouse screening project for HL at the Jackson Laboratory in the USA (Zheng et al. 1999) and the ENU mutagenesis initiative at the MRC and Wellcome Trust Sanger Institute in the UK (Hardisty et al. 1999), have provided valuable information from mouse models of HL. These data allowed rapid identification and validation of candidate human genes in a specific mapped region, adding to the repertoire of total genes with a role in hearing and its aberration. Last but not the least, The Human Genome Project has been a major factor which catapulted these discoveries at a much faster pace. As of 2016, a total 169 loci have been mapped for nonsyndromic forms of HL (Hereditary Hearing Loss home page), and 85 of these genes have been identified (Jasper et al. 2015). An additional 42 genes have been identified for syndromic forms of hearing loss, making the total number daunting when it comes to offering diagnostic testing. This recent explosion in gene discovery for HL can also be attributed to technological advances in massively parallel sequencing (MPS) also referred to as Next Generation Sequencing (NGS). Between 1995 and 2010, nearly 75% of the genes were identified using more traditional methods; however, since 2010 another 25% of genes have been identified in a short span, emphasizing the impact of these technologies in enhancing gene discovery (Atik et al. 2015). Although the initial identification of genes such as *TRPN* for DFNB79 relied on homozygosity mapping to filter whole exome sequencing data (Rehman, 2010), more recent reports of genes such as *EPS8* and *FAM65B* identified in consanguineous families with HL had no prior mapping studies to guide the search (Behlouli et al. 2014; Diaz‐Horta et al. 2014). This sudden explosion in our knowledge of genes important for hearing has guided functional studies in animal models, to elucidate the cell biology and auditory physiology (Dror and Avraham 2009; Richardson et al. 2011). For example, we can now better understand the architecture of the hearing apparatus, with genes involved in the cytoskeleton including the actin‐rich stereocilia in the hair cell bundles, such as myosins (*MYO7A*,* MYO6*,* MYO3A*,* MYO1A*, and others), cell‐cell junctions (includes gap‐junction proteins such as connexin‐26 and connexin‐30 and others), membrane transporters such as *SLC26A4* encoding the pendrin protein and the other potassium channels (*KCNQ4*) as well as regulatory elements including several transcription factors. The discovery of various genes, mutations in which can cause Usher syndrome, have helped elucidate the molecular mechanisms underlying hair bundle development and mechano‐electrical transduction (Richardson et al. 2011).

## Early Hearing Detection and Intervention (EHDI) Programs

Another major milestone in this journey of understanding the etiology and mechanisms of hearing loss has been the introduction and implementation of universal audiologic newborn hearing screening in the United States and several other Western nations. Since its introduction in early 2000 in a select few states, it is now adopted by all states in the USA (NCHAM website). The advent of newborn hearing screening has made it possible to detect HL by 2–3 months of age, and infants who begin rehabilitation by 6 months of age have much improved language and scholastic outcomes compared to those whose HL is detected after 6 months of age (Yoshinaga‐Itano 2003). With improved technology and education, the EHDI programs have been successful in decreasing the false‐positive rates, and have improved their loss to follow‐up of infants identified at birth for confirmatory testing (Smith et al. 2014). Despite the tremendous success of these programs, there remain the issues of missing select forms of congenital HL which do not present at birth such as due to congenital CMV infections, Pendred syndrome, and the late onset dominant forms of HL. We have also shown that a small percent of neonates with GJB2 HL are missed by audiologic newborn screening (Norris et al. 2006). In response to these limitations, there has been a push to add etiologic diagnosis via molecular testing in newborns although this has not been implemented in clinical practice (Gardner et al. 2006; Giersch et al. 2016). Another major impetus to having an etiologic diagnosis for the HL has been the promise of newer therapies shown to have benefits in mouse models which are specific for a genetic defect as discussed below. Unfortunately, despite the recommendations of professional organizations (Professional Practice and Guidelines Committee 2014) and the Joint Commission on Infant Hearing, systematic genetic evaluation and counseling are still not a standard practice for infants confirmed with HL through screening.

## Advances in Etiologic Diagnosis of Hearing Loss

Although the pace of discovery of genes for both nonsyndromic and syndromic HL has been phenomenal, the extreme genetic heterogeneity has precluded comprehensive genetic testing that is cost effective and easily accessible. Until a few years ago, testing was limited to the more common genetic forms of HL including the GJB2, GJB6, and select mitochondrial genes. This no doubt was helpful in those families where a pathogenic mutation was identified. These results helped stop the diagnostic odyssey, offer precise genetic counseling and recurrence risks, and prevent onset of HL if a mitochondrial A1555G change was detected prior to administration of aminoglycoside antibiotics (Pandya and Arnos 2006). With the advent and availability of technology using massively parallel DNA sequencing, it is now possible to interrogate most genes identified for syndromic and nonsyndromic hearing loss using large gene panels (Shearer et al. 2011). Currently, such platforms and panels are offered through a few laboratories in the USA; however, there remains variability in the number and types of genes included on these panels (Jasper et al. 2015). Additionally, prospective studies and literature review to assess the sensitivity of panels using MPS suggest an overall diagnostic rate of 42%, with higher values if an individual has bilateral AR NSHL versus milder forms of HL (Shearer et al. 2013; Shearer and Smith 2015). No doubt, the current platforms of gene panels have facilitated dramatic improvements in the diagnostic yield, but there remains the need for HL diagnostic panels which are more comprehensive that include genes implicated in both syndromic and nonsyndromic HL. Although one can consider using whole exome sequencing (WES) in lieu of using targeted gene panels for HL, considerations favoring the latter approach include the high cost, and the identification of secondary findings unrelated to the presenting phenotype with WES (Shearer et al. 2013). Not only does having an etiologic diagnosis for HL provide knowledge about the natural history, progression of HL, associated organ system involvement, and precise recurrence risk, but in the near future it will form the basis for novel gene‐ or mutation‐specific treatment options to ameliorate or treat genetic HL.

## Advances in Management of Hearing Loss

Despite the significant progress made in the last decade in our understanding of the causative genes which result in hearing loss, the cornerstone of treatment for deafness remains nonspecific, irrespective of the etiology. The main treatment modalities available include the use of hearing aids for individuals with residual hearing and cochlear implantation in those with severe to profound hearing loss. Both forms of therapy have come a long way with significant technological advances. Cochlear implants (CI) are now offered to infants and placed in both ears to offer the maximal benefit. However, management approaches and options for sensorineural hearing loss continue to lag behind the significant understanding of the basic genetic defects causing HL. The past few years have seen a glimmer of hope for treatments focused on the basic genetic defect for HL, albeit in animal models. Hearing restoration to near normal was demonstrated in a gene therapy study which reinstated the expression of vesicular glutamate transporter 3 (VGLUT3) using postnatal AAV‐mediated delivery (Akil et al. 2012). Askew et al. (2015) demonstrated restoration of the mechano‐transducer current and partial hearing in mice, with transmembrane channel‐like 1 and 2 (TMC‐1 and TMC‐2) gene constructs using AAV vectors. Antisense oligonucleotide treatment injected intraperitoneally against a splicing mutation in Usher Syndrome Type I C mouse model (Ush1c216AA knock‐in mice) ameliorated auditory function (Lentz et al. 2013). Therapeutic approaches beyond gene replacement or transcript correction are slightly further along as with a FDA‐approved clinical trial to use adenovirus for ectopic expression of AtoH1, a transcription factor, in cochlear nonsensory cells (Clinical Trials.gov identifier: NCT02132130). Despite the progress in these therapeutic approaches, there remain several key questions about the optimal timing for such a therapeutic approach – should it be at the time when the gene is expressed, or when the phenotype is manifest, and determining the location for introduction of a gene of interest (Minoda et al. 2015)

## Future Directions

This is an exciting time for those involved in the field of hearing loss, given the explosion in our understanding of the molecular basis of deafness, and the mechanisms and function leading to hearing loss. The implementation of audiologic newborn screening allows early diagnosis of HL with appropriate interventions to prevent language delay. However, as we have got a glimpse into the future of specific gene‐ and mutation‐based therapeutics for HL, it becomes imperative that we focus our attention on enhancing the EHDI programs with complementary molecular diagnosis in newborns for an etiologic diagnosis. Until we determine a specific etiologic diagnosis for the HL, offering timely gene‐based therapeutics may not be feasible. In contrast, it is possible that modalities for hair cell regeneration may not need a specific etiologic diagnosis. However, based on experience with newborn metabolic screening, we have learnt that understanding the natural history and having a specific diagnosis are crucial to implementing state of the art patient care and therapies. There is also a need for additional research to understand the molecular basis for the auditory neuropathy spectrum disorders as well as age‐related hearing loss in the future.
